# 14-Meth­oxy-4,6-dimethyl-9-phenyl-8,12-dioxa-4,6-di­aza­tetra­cyclo­[8.8.0.0^2,7^.0^13,18^]octa­deca-2(7),13,15,17-tetra­ene-3,5,11-trione

**DOI:** 10.1107/S1600536813017789

**Published:** 2013-07-03

**Authors:** G Jagadeesan, S. Jayashree, D. Kannan, M. Bakthadoss, S. Aravindhan

**Affiliations:** a Department of Physics, Presidency College, Chennai 600 005, India; b Center for Advanced Study in Botany, University of Madras, Guindy Campus, Chennai 600 025, India; c Department of Organic Chemistry, University of Madras, Guindy Campus, Chennai 600 025, India

## Abstract

The title compound, C_23_H_20_N_2_O_6_, crystallizes with two mol­ecules in the asymmetric unit in which the dihedral angles between the mean planes of the pyran and phenyl rings are 66.6 (1) and 61.9 (1) °. The fused pyrone and pyran rings each adopts a sofa conformation. In the crystal, C—H⋯O hydrogen bonds link the mol­ecules, forming a two-dimensional network parallel to [001].

## Related literature

For the biological activity of pyran­ocoumarin compounds, see: Kawaii *et al.* (2001 ▶); Hossain *et al.* (1996 ▶); Goel *et al.* (1997 ▶); Su *et al.* (2009 ▶); Xu *et al.* (2006 ▶). For anti-filarial activity studies of pyran­ocoumarin compounds, see: Casley-Smith *et al.* (1993 ▶) and for enzyme inhibitory activity of pyran­ocoumarin compounds, see: Pavao *et al.* (2002 ▶). For a related structure, see: Jagadeesan *et al.* (2013 ▶).
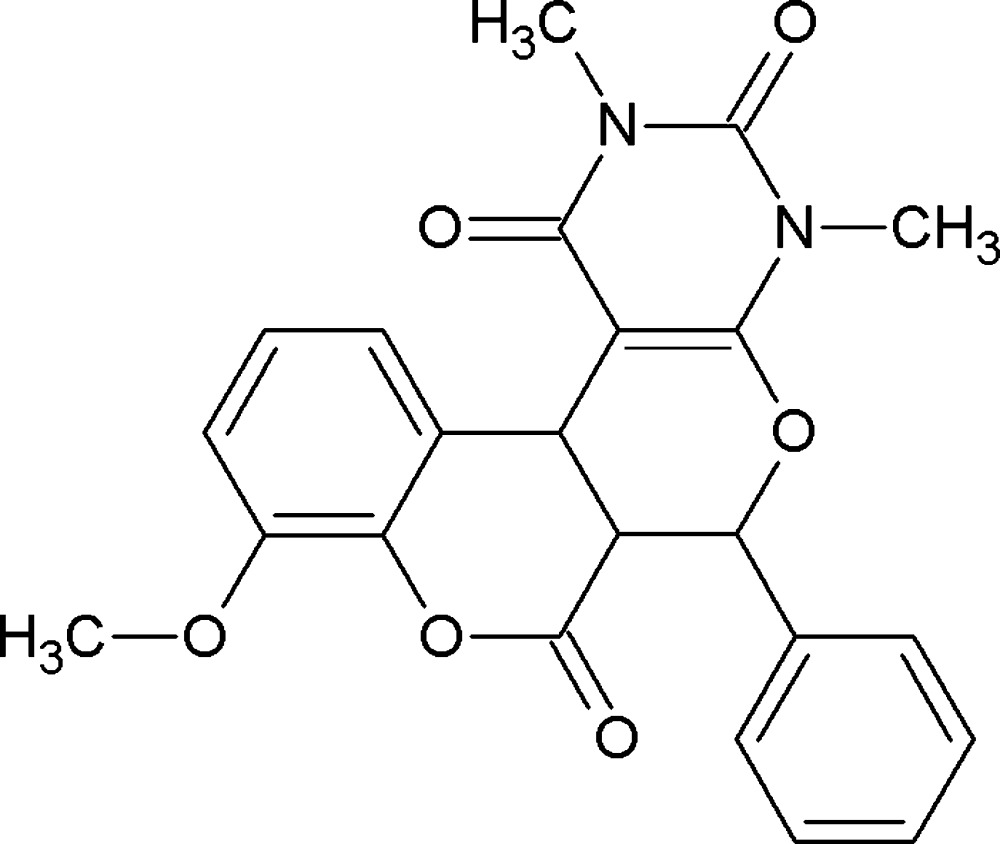


## Experimental

### 

#### Crystal data

C_23_H_20_N_2_O_6_
*M* *_r_* = 420.41
Triclinic, 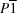

*a* = 9.2283 (7) Å
*b* = 14.0584 (10) Å
*c* = 16.4457 (13) Å
α = 94.869 (2)°
β = 102.547 (2)°
γ = 105.270 (2)°
*V* = 1986.1 (3) Å^3^
*Z* = 4
Mo *K*α radiation
μ = 0.10 mm^−1^
*T* = 293 K
0.25 × 0.20 × 0.20 mm

#### Data collection

Bruker Kappa APEXII CCD diffractometer
Absorption correction: multi-scan (*SADABS*; Bruker 2004 ▶) *T*_min_ = 0.979, *T*_max_ = 0.983
36657 measured reflections
7558 independent reflections
5648 reflections with *I* > 2σ(*I*)
*R*_int_ = 0.029

#### Refinement

*R*[*F* ^2^ > 2σ(*F* ^2^)] = 0.047
*wR*(*F* ^2^) = 0.141
*S* = 1.06
7558 reflections
588 parameters
H atoms treated by a mixture of independent and constrained refinement
Δρ_max_ = 0.22 e Å^−3^
Δρ_min_ = −0.18 e Å^−3^

### 

Data collection: *APEX2* (Bruker, 2004 ▶); cell refinement: *APEX2* and *SAINT* (Bruker, 2004 ▶); data reduction: *SAINT* and *XPREP* (Bruker, 2004 ▶); program(s) used to solve structure: *SHELXS97* (Sheldrick, 2008 ▶); program(s) used to refine structure: *SHELXL97* (Sheldrick, 2008 ▶); molecular graphics: *ORTEP-3 for Windows* (Farrugia, 2012 ▶); software used to prepare material for publication: *PLATON* (Spek, 2009 ▶).

## Supplementary Material

Crystal structure: contains datablock(s) I, global. DOI: 10.1107/S1600536813017789/bt6915sup1.cif

Structure factors: contains datablock(s) I. DOI: 10.1107/S1600536813017789/bt6915Isup2.hkl

Supplementary material file. DOI: 10.1107/S1600536813017789/bt6915Isup3.cml

Additional supplementary materials:  crystallographic information; 3D view; checkCIF report

## Figures and Tables

**Table 1:** Hydrogen-bond geometry (Å, °)

*D*—H⋯*A*	*D*—H	H⋯*A*	*D*⋯*A*	*D*—H⋯*A*
C21*A*—H21*A*⋯O5*B* ^i^	0.93	2.55	3.435 (4)	159
C20*B*—H20*B*⋯O5*A* ^ii^	0.93	2.34	3.175 (3)	149
C23*B*—H23*D*⋯O6*B* ^iii^	0.96	2.46	3.249 (4)	139

Symmetry codes: (i) 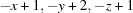; (ii) 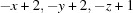; (iii) 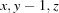.

**Fig. 1.**
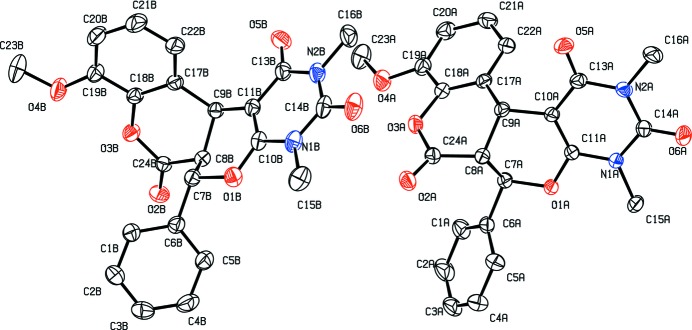
The molecular structure of the title compound. Displacement ellipsoids are drawn at the 30% probability level, H atoms have been omitted for clarity.

**Fig. 2.**
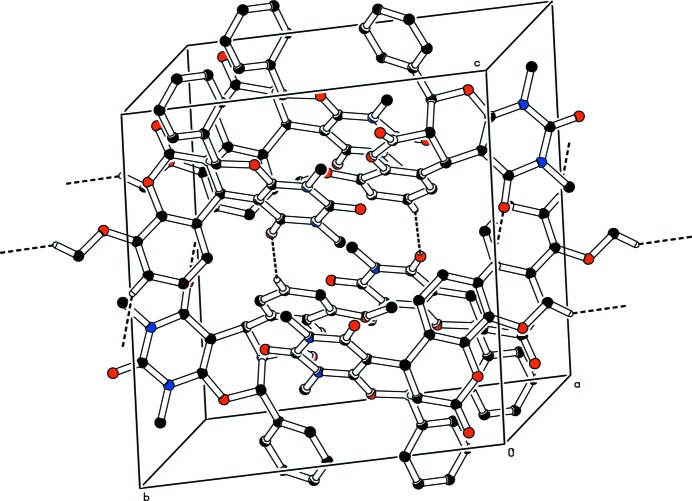
Crystal packing of the title compound. Hydrogen bonds are shown as dashed lines. For the sake of clarity, H atoms not involved in the interactions have been omitted.

## supplementary crystallographic information

### Comment

Coumarin derivatives show strong activity against cancer cell lines (Kawaii *et al.*, 2001) and exhibit monoamine oxidase inhibitory activity (Hossain *et al.*, 1996). Antiulcer activity of some naturally occurring pyranocoumarins has been reported (Goel *et al.*, 1997). They also show anti-hepatitis B virus, anti-filarial (Casley-Smith *et al.*, 1993) and cytotoxic activities (Su *et al.*, 2009) and anti-TB activity (Xu *et al.*, 2006). One natural source coumarin derivative, Chalepin, inhibits the glyceraldehyde-3-phosphate dehydrogenase of parasites (Protein Data Bank ID code 1 K3T) (Pavao *et al.*, 2002). Herein, we report on the crystal structure of the title coumarin derivative.

We have already reported a similar compound (Jagadeesan *et al.*, 2013). The title compound crystallizes with two molecules in the asymmetric unit. Geometrical parameters are almost similar the title compound and the previously reported structure. The six-membered pyrone and pyran rings adopt a sofa conformation. The dihedral angle between the mean planes of the pyran and phenyl rings is 66.6 (1) ° and 61.9 (1) ° for the molecule A and B respectively. The crystal packing is stabilized by C—H···O intermolecular hydrogen bonds (Fig. 2 and Table 1).

### Experimental

A mixture of 2-ethoxy-6-formylphenyl (2E)-but-2-enoate (0.234 g, 1 mmol) and *N*,*N*-dimethylbarbituric acid (0.156 g, 1 mmol) was placed in a round bottom flask and melted at 180 °C for 1 h. After completion of the reaction as indicated by TLC, the crude product was washed with 5 ml of ethylacetate and hexane mixture (1:49 ratio) which successfully provided the pure product in 90% yield as colorless solid. Diffraction quality crystals were obtained by slow evaporation of a solution in (Methonal and ethanol)6:4 ratio.

### Refinement

H atoms (except H7A, H7B, H8A, H8B, H9A and H9B atom which were freely refined) were refined with fixed individual displacement parameters [U(H) = 1.2 *U*_eq_(C)] using a riding model with C—H ranging from 0.93 Å to 0.97 Å. The methyl groups bonded to N were refined as disordered over two equally occupied sites rotated by 60 degrees.

### Crystal data

**Table:**

C_23_H_20_N_2_O_6_	*Z* = 4
*M**_r_* = 420.41	*F*(000) = 880
Triclinic, *P*1	*D*_x_ = 1.406 Mg m^−^^3^
Hall symbol: -P 1	Mo *K*α radiation, λ = 0.71073 Å
*a* = 9.2283 (7) Å	Cell parameters from 8834 reflections
*b* = 14.0584 (10) Å	θ = 2.1–31.2°
*c* = 16.4457 (13) Å	µ = 0.10 mm^−^^1^
α = 94.869 (2)°	*T* = 293 K
β = 102.547 (2)°	Block, colourless
γ = 105.270 (2)°	0.25 × 0.20 × 0.20 mm
*V* = 1986.1 (3) Å^3^	

### Data collection

**Table:**

Bruker Kappa APEXII CCD diffractometer	7558 independent reflections
Radiation source: fine-focus sealed tube	5648 reflections with *I* > 2σ(*I*)
Graphite monochromator	*R*_int_ = 0.029
ω and φ scan	θ_max_ = 25.7°, θ_min_ = 1.3°
Absorption correction: multi-scan (*SADABS*; Bruker 2004)	*h* = −11→11
*T*_min_ = 0.979, *T*_max_ = 0.983	*k* = −17→17
36657 measured reflections	*l* = −20→20

### Refinement

**Table:**

Refinement on *F*^2^	Secondary atom site location: difference Fourier map
Least-squares matrix: full	Hydrogen site location: inferred from neighbouring sites
*R*[*F*^2^ > 2σ(*F*^2^)] = 0.047	H atoms treated by a mixture of independent and constrained refinement
*wR*(*F*^2^) = 0.141	*w* = 1/[σ^2^(*F*_o_^2^) + (0.0528*P*)^2^ + 1.2155*P*] where *P* = (*F*_o_^2^ + 2*F*_c_^2^)/3
*S* = 1.06	(Δ/σ)_max_ = 0.001
7558 reflections	Δρ_max_ = 0.22 e Å^−^^3^
588 parameters	Δρ_min_ = −0.18 e Å^−^^3^
0 restraints	Extinction correction: *SHELXL97* (Sheldrick, 2008), Fc^*^=kFc[1+0.001xFc^2^λ^3^/sin(2θ)]^-1/4^
Primary atom site location: structure-invariant direct methods	Extinction coefficient: 0.0041 (7)

### Special details

**Table:**

Geometry. All e.s.d.'s (except the e.s.d. in the dihedral angle between two l.s. planes) are estimated using the full covariance matrix. The cell e.s.d.'s are taken into account individually in the estimation of e.s.d.'s in distances, angles and torsion angles; correlations between e.s.d.'s in cell parameters are only used when they are defined by crystal symmetry. An approximate (isotropic) treatment of cell e.s.d.'s is used for estimating e.s.d.'s involving l.s. planes.
Refinement. Refinement of *F*^2^ against ALL reflections. The weighted *R*-factor *wR* and goodness of fit *S* are based on *F*^2^, conventional *R*-factors *R* are based on *F*, with *F* set to zero for negative *F*^2^. The threshold expression of *F*^2^ > σ(*F*^2^) is used only for calculating *R*-factors(gt) *etc*. and is not relevant to the choice of reflections for refinement. *R*-factors based on *F*^2^ are statistically about twice as large as those based on *F*, and *R*- factors based on ALL data will be even larger.

### Fractional atomic coordinates and isotropic or equivalent isotropic displacement parameters (Å^2^)

**Table:**

	*x*	*y*	*z*	*U*_iso_*/*U*_eq_	Occ. (<1)
O1A	0.43958 (18)	0.84184 (11)	0.13771 (9)	0.0433 (4)	
O3A	0.62793 (19)	0.60615 (11)	0.25301 (10)	0.0491 (4)	
N1A	0.4243 (2)	0.98731 (13)	0.19611 (11)	0.0408 (4)	
O6A	0.3970 (2)	1.13287 (12)	0.25056 (11)	0.0653 (5)	
O5A	0.6491 (2)	0.96419 (12)	0.42706 (10)	0.0587 (5)	
O1B	1.0081 (2)	1.35732 (12)	0.17540 (11)	0.0542 (4)	
O2B	0.7090 (2)	1.04069 (13)	0.08928 (10)	0.0558 (4)	
O3B	0.8419 (2)	1.03414 (11)	0.21441 (10)	0.0500 (4)	
O2A	0.7740 (2)	0.66004 (13)	0.16873 (11)	0.0553 (4)	
C11A	0.4727 (2)	0.90470 (15)	0.20928 (13)	0.0354 (5)	
N2A	0.5173 (2)	1.04671 (13)	0.34024 (11)	0.0447 (5)	
C9A	0.6168 (3)	0.80548 (15)	0.29473 (13)	0.0370 (5)	
C10A	0.5470 (3)	0.89086 (15)	0.28534 (13)	0.0371 (5)	
C24A	0.6857 (3)	0.67602 (16)	0.20697 (14)	0.0417 (5)	
C17A	0.5294 (3)	0.71943 (15)	0.33133 (13)	0.0381 (5)	
O4A	0.4761 (2)	0.45275 (12)	0.30578 (12)	0.0642 (5)	
N1B	1.0045 (2)	1.49711 (14)	0.25095 (14)	0.0557 (5)	
O5B	0.7679 (2)	1.34929 (14)	0.40134 (12)	0.0643 (5)	
C14A	0.4427 (3)	1.06042 (15)	0.26200 (14)	0.0439 (5)	
C6A	0.4536 (3)	0.69790 (17)	0.05832 (14)	0.0436 (5)	
C11B	0.9629 (3)	1.39480 (16)	0.23985 (15)	0.0453 (6)	
N2B	0.8912 (3)	1.49558 (14)	0.36477 (13)	0.0540 (5)	
C8B	0.8289 (3)	1.19799 (16)	0.17843 (14)	0.0407 (5)	
C18A	0.5376 (3)	0.62478 (16)	0.30704 (14)	0.0400 (5)	
C10B	0.8851 (3)	1.34072 (16)	0.28866 (14)	0.0424 (5)	
C18B	0.9296 (3)	1.08267 (16)	0.29447 (14)	0.0424 (5)	
O4B	1.0043 (2)	0.93660 (13)	0.30200 (13)	0.0700 (6)	
C22A	0.4416 (3)	0.72982 (17)	0.38821 (14)	0.0470 (6)	
H22A	0.4321	0.7923	0.4049	0.056*	
C13B	0.8415 (3)	1.39109 (17)	0.35471 (16)	0.0489 (6)	
C8A	0.6299 (3)	0.76749 (16)	0.20753 (14)	0.0382 (5)	
C24B	0.7878 (3)	1.08628 (16)	0.15531 (14)	0.0411 (5)	
C9B	0.8319 (3)	1.22862 (15)	0.26978 (14)	0.0397 (5)	
C7B	0.9863 (3)	1.25026 (17)	0.16340 (15)	0.0450 (5)	
C19A	0.4606 (3)	0.54112 (16)	0.33674 (15)	0.0469 (6)	
C17B	0.9294 (3)	1.17652 (16)	0.32621 (14)	0.0421 (5)	
O6B	1.0114 (2)	1.64095 (13)	0.32677 (14)	0.0782 (6)	
C13A	0.5775 (3)	0.96662 (16)	0.35610 (14)	0.0423 (5)	
C7A	0.4712 (3)	0.74697 (16)	0.14575 (14)	0.0394 (5)	
C14B	0.9705 (3)	1.55068 (17)	0.31511 (18)	0.0560 (7)	
C6B	1.0041 (3)	1.22284 (18)	0.07678 (15)	0.0470 (6)	
C21A	0.3684 (3)	0.64850 (19)	0.42018 (16)	0.0552 (6)	
H21A	0.3124	0.6570	0.4595	0.066*	
C15A	0.3540 (3)	1.00239 (18)	0.11075 (14)	0.0508 (6)	
H15A	0.2965	1.0497	0.1143	0.076*	0.50
H15B	0.2856	0.9401	0.0795	0.076*	0.50
H15C	0.4342	1.0272	0.0827	0.076*	0.50
H15D	0.3810	0.9616	0.0700	0.076*	0.50
H15E	0.3919	1.0713	0.1049	0.076*	0.50
H15F	0.2433	0.9841	0.1017	0.076*	0.50
C2B	1.0921 (3)	1.1218 (2)	−0.01454 (18)	0.0654 (7)	
H2B	1.1418	1.0733	−0.0220	0.079*	
C16A	0.5385 (4)	1.12317 (18)	0.41195 (16)	0.0638 (8)	
H16A	0.5481	1.0942	0.4629	0.096*	0.50
H16B	0.4505	1.1488	0.4037	0.096*	0.50
H16C	0.6307	1.1765	0.4160	0.096*	0.50
H16D	0.5381	1.1855	0.3922	0.096*	0.50
H16E	0.6357	1.1309	0.4514	0.096*	0.50
H16F	0.4555	1.1031	0.4391	0.096*	0.50
C1A	0.3713 (3)	0.59853 (19)	0.03436 (17)	0.0594 (7)	
H1A	0.3277	0.5627	0.0725	0.071*	
C5B	0.9430 (4)	1.2624 (2)	0.00862 (18)	0.0640 (7)	
H5B	0.8912	1.3098	0.0157	0.077*	
C20A	0.3767 (3)	0.55408 (18)	0.39469 (16)	0.0547 (6)	
H20A	0.3261	0.4996	0.4165	0.066*	
C20B	1.1029 (4)	1.0712 (2)	0.42048 (17)	0.0660 (8)	
H20B	1.1602	1.0364	0.4531	0.079*	
C19B	1.0153 (3)	1.02850 (18)	0.34063 (16)	0.0510 (6)	
C16B	0.8547 (4)	1.5509 (2)	0.43332 (19)	0.0728 (9)	
H16G	0.8232	1.5067	0.4718	0.109*	0.50
H16H	0.7721	1.5781	0.4105	0.109*	0.50
H16I	0.9450	1.6041	0.4626	0.109*	0.50
H16J	0.8703	1.6192	0.4248	0.109*	0.50
H16K	0.9214	1.5478	0.4861	0.109*	0.50
H16L	0.7486	1.5218	0.4340	0.109*	0.50
C22B	1.0194 (4)	1.2177 (2)	0.40600 (16)	0.0621 (7)	
H22B	1.0219	1.2812	0.4288	0.075*	
C23A	0.3997 (4)	0.36508 (19)	0.3349 (2)	0.0740 (9)	
H23A	0.4203	0.3082	0.3084	0.111*	
H23B	0.2900	0.3563	0.3209	0.111*	
H23C	0.4374	0.3717	0.3949	0.111*	
C4A	0.4996 (4)	0.7023 (3)	−0.07899 (17)	0.0719 (8)	
H4A	0.5437	0.7374	−0.1173	0.086*	
C3A	0.4174 (4)	0.6034 (3)	−0.10228 (19)	0.0758 (10)	
H3A	0.4055	0.5715	−0.1563	0.091*	
C2A	0.3535 (4)	0.5522 (2)	−0.0460 (2)	0.0766 (9)	
H2A	0.2973	0.4853	−0.0619	0.092*	
C5A	0.5171 (3)	0.7500 (2)	0.00085 (16)	0.0579 (7)	
H5A	0.5717	0.8173	0.0160	0.069*	
C1B	1.0790 (3)	1.1524 (2)	0.06444 (17)	0.0595 (7)	
H1B	1.1214	1.1251	0.1101	0.071*	
C3B	1.0330 (3)	1.1619 (2)	−0.08186 (19)	0.0670 (8)	
H3B	1.0434	1.1417	−0.1351	0.080*	
C23B	1.0990 (4)	0.8826 (2)	0.3457 (2)	0.0904 (11)	
H23D	1.0807	0.8192	0.3123	0.136*	
H23E	1.0739	0.8723	0.3986	0.136*	
H23F	1.2062	0.9198	0.3557	0.136*	
C15B	1.0871 (4)	1.5530 (2)	0.1958 (2)	0.0786 (9)	
H15G	1.1215	1.6227	0.2182	0.118*	0.50
H15H	1.0190	1.5428	0.1406	0.118*	0.50
H15I	1.1752	1.5301	0.1922	0.118*	0.50
H15J	1.0889	1.5077	0.1491	0.118*	0.50
H15K	1.1915	1.5876	0.2268	0.118*	0.50
H15L	1.0353	1.6003	0.1752	0.118*	0.50
C21B	1.1056 (4)	1.1655 (2)	0.45203 (18)	0.0757 (9)	
H21B	1.1670	1.1946	0.5056	0.091*	
C4B	0.9580 (4)	1.2323 (2)	−0.07059 (19)	0.0745 (9)	
H4B	0.9169	1.2599	−0.1164	0.089*	
H8A	0.703 (3)	0.8181 (16)	0.1882 (13)	0.040 (6)*	
H9A	0.723 (3)	0.8307 (15)	0.3302 (13)	0.036 (6)*	
H7A	0.393 (3)	0.7075 (17)	0.1685 (14)	0.041 (6)*	
H7B	1.071 (3)	1.2327 (17)	0.2088 (15)	0.050 (6)*	
H9B	0.724 (3)	1.2058 (16)	0.2746 (14)	0.043 (6)*	
H8B	0.750 (3)	1.2190 (17)	0.1387 (15)	0.048 (6)*	

### Atomic displacement parameters (Å^2^)

**Table:**

	*U*^11^	*U*^22^	*U*^33^	*U*^12^	*U*^13^	*U*^23^
O1A	0.0583 (10)	0.0410 (8)	0.0318 (8)	0.0254 (7)	0.0018 (7)	0.0004 (6)
O3A	0.0613 (10)	0.0405 (8)	0.0584 (10)	0.0253 (7)	0.0263 (9)	0.0144 (7)
N1A	0.0528 (11)	0.0378 (9)	0.0348 (10)	0.0206 (8)	0.0080 (8)	0.0063 (7)
O6A	0.1003 (15)	0.0422 (9)	0.0583 (11)	0.0361 (10)	0.0117 (10)	0.0054 (8)
O5A	0.0825 (13)	0.0518 (10)	0.0337 (9)	0.0201 (9)	−0.0012 (9)	0.0015 (7)
O1B	0.0591 (11)	0.0416 (9)	0.0684 (12)	0.0125 (8)	0.0287 (9)	0.0163 (8)
O2B	0.0619 (11)	0.0555 (10)	0.0412 (10)	0.0129 (8)	0.0046 (8)	−0.0052 (8)
O3B	0.0636 (11)	0.0352 (8)	0.0442 (9)	0.0123 (7)	0.0018 (8)	0.0038 (7)
O2A	0.0584 (11)	0.0557 (10)	0.0633 (11)	0.0266 (8)	0.0267 (9)	0.0097 (8)
C11A	0.0394 (12)	0.0331 (10)	0.0338 (11)	0.0117 (9)	0.0084 (9)	0.0037 (8)
N2A	0.0644 (13)	0.0330 (9)	0.0360 (10)	0.0129 (8)	0.0140 (9)	0.0017 (7)
C9A	0.0395 (12)	0.0371 (11)	0.0322 (11)	0.0126 (9)	0.0029 (10)	0.0042 (9)
C10A	0.0457 (12)	0.0328 (10)	0.0334 (11)	0.0119 (9)	0.0099 (9)	0.0057 (8)
C24A	0.0420 (13)	0.0429 (12)	0.0419 (13)	0.0159 (10)	0.0097 (11)	0.0057 (10)
C17A	0.0434 (12)	0.0392 (11)	0.0307 (11)	0.0135 (9)	0.0041 (9)	0.0073 (9)
O4A	0.0877 (14)	0.0372 (9)	0.0766 (13)	0.0218 (9)	0.0317 (11)	0.0158 (8)
N1B	0.0546 (13)	0.0355 (10)	0.0732 (15)	0.0094 (9)	0.0105 (11)	0.0137 (10)
O5B	0.0829 (14)	0.0570 (11)	0.0583 (11)	0.0210 (10)	0.0300 (10)	0.0032 (9)
C14A	0.0569 (14)	0.0314 (11)	0.0438 (13)	0.0122 (10)	0.0143 (11)	0.0051 (9)
C6A	0.0476 (13)	0.0443 (12)	0.0392 (12)	0.0220 (10)	0.0029 (10)	0.0004 (10)
C11B	0.0420 (13)	0.0385 (12)	0.0542 (15)	0.0133 (10)	0.0068 (11)	0.0076 (10)
N2B	0.0594 (13)	0.0400 (11)	0.0569 (13)	0.0198 (9)	0.0004 (10)	−0.0034 (9)
C8B	0.0435 (13)	0.0410 (12)	0.0391 (12)	0.0142 (10)	0.0096 (10)	0.0096 (9)
C18A	0.0443 (13)	0.0411 (12)	0.0382 (12)	0.0178 (10)	0.0098 (10)	0.0093 (9)
C10B	0.0440 (13)	0.0357 (11)	0.0459 (13)	0.0124 (9)	0.0069 (10)	0.0058 (9)
C18B	0.0495 (13)	0.0365 (11)	0.0389 (12)	0.0098 (9)	0.0088 (10)	0.0072 (9)
O4B	0.0868 (14)	0.0456 (10)	0.0782 (13)	0.0314 (9)	0.0049 (11)	0.0136 (9)
C22A	0.0609 (15)	0.0447 (13)	0.0382 (13)	0.0185 (11)	0.0145 (11)	0.0062 (10)
C13B	0.0514 (14)	0.0429 (13)	0.0497 (14)	0.0180 (11)	0.0035 (12)	0.0009 (11)
C8A	0.0405 (12)	0.0379 (11)	0.0390 (12)	0.0141 (9)	0.0111 (10)	0.0084 (9)
C24B	0.0439 (13)	0.0417 (12)	0.0384 (13)	0.0116 (10)	0.0130 (10)	0.0057 (10)
C9B	0.0442 (13)	0.0353 (11)	0.0386 (12)	0.0100 (9)	0.0100 (10)	0.0060 (9)
C7B	0.0470 (14)	0.0441 (12)	0.0483 (14)	0.0171 (10)	0.0141 (11)	0.0124 (10)
C19A	0.0538 (14)	0.0383 (12)	0.0488 (14)	0.0147 (10)	0.0099 (11)	0.0108 (10)
C17B	0.0497 (13)	0.0393 (12)	0.0372 (12)	0.0117 (10)	0.0110 (10)	0.0084 (9)
O6B	0.0784 (14)	0.0347 (10)	0.1059 (17)	0.0141 (9)	−0.0036 (12)	0.0029 (10)
C13A	0.0521 (14)	0.0356 (11)	0.0369 (12)	0.0085 (9)	0.0108 (10)	0.0068 (9)
C7A	0.0459 (13)	0.0361 (11)	0.0363 (12)	0.0151 (10)	0.0074 (10)	0.0036 (9)
C14B	0.0517 (15)	0.0361 (13)	0.0696 (18)	0.0162 (11)	−0.0089 (13)	0.0015 (12)
C6B	0.0453 (13)	0.0522 (13)	0.0522 (14)	0.0183 (11)	0.0202 (11)	0.0200 (11)
C21A	0.0680 (17)	0.0575 (15)	0.0455 (14)	0.0183 (13)	0.0237 (13)	0.0110 (11)
C15A	0.0648 (16)	0.0473 (13)	0.0412 (13)	0.0262 (12)	0.0018 (11)	0.0093 (10)
C2B	0.0679 (19)	0.080 (2)	0.0646 (18)	0.0361 (15)	0.0311 (15)	0.0143 (15)
C16A	0.105 (2)	0.0425 (14)	0.0419 (14)	0.0209 (14)	0.0193 (14)	−0.0039 (11)
C1A	0.0709 (18)	0.0472 (14)	0.0573 (16)	0.0205 (12)	0.0111 (14)	−0.0049 (12)
C5B	0.0758 (19)	0.0689 (18)	0.0648 (18)	0.0357 (15)	0.0288 (15)	0.0288 (14)
C20A	0.0630 (16)	0.0463 (14)	0.0544 (15)	0.0099 (11)	0.0176 (13)	0.0168 (11)
C20B	0.077 (2)	0.0672 (18)	0.0556 (17)	0.0316 (15)	0.0022 (14)	0.0217 (14)
C19B	0.0570 (15)	0.0427 (13)	0.0544 (15)	0.0164 (11)	0.0106 (12)	0.0150 (11)
C16B	0.089 (2)	0.0595 (17)	0.0654 (18)	0.0384 (16)	−0.0015 (16)	−0.0133 (14)
C22B	0.087 (2)	0.0553 (15)	0.0397 (14)	0.0265 (14)	0.0031 (13)	−0.0002 (11)
C23A	0.084 (2)	0.0372 (14)	0.098 (2)	0.0115 (13)	0.0205 (18)	0.0200 (14)
C4A	0.094 (2)	0.090 (2)	0.0437 (16)	0.0452 (19)	0.0195 (15)	0.0082 (15)
C3A	0.098 (2)	0.090 (2)	0.0429 (16)	0.055 (2)	0.0001 (16)	−0.0169 (16)
C2A	0.090 (2)	0.0618 (18)	0.068 (2)	0.0281 (16)	0.0031 (18)	−0.0222 (16)
C5A	0.0720 (18)	0.0593 (15)	0.0439 (15)	0.0230 (13)	0.0154 (13)	0.0014 (12)
C1B	0.0658 (17)	0.0744 (18)	0.0565 (16)	0.0391 (14)	0.0254 (14)	0.0213 (13)
C3B	0.0686 (19)	0.082 (2)	0.0587 (18)	0.0176 (15)	0.0343 (15)	0.0174 (15)
C23B	0.096 (3)	0.0618 (19)	0.123 (3)	0.0421 (18)	0.015 (2)	0.0345 (19)
C15B	0.085 (2)	0.0473 (16)	0.101 (2)	0.0049 (14)	0.0274 (19)	0.0289 (16)
C21B	0.098 (2)	0.076 (2)	0.0435 (15)	0.0330 (17)	−0.0105 (15)	0.0042 (14)
C4B	0.091 (2)	0.084 (2)	0.0600 (19)	0.0308 (18)	0.0271 (17)	0.0381 (16)

### Geometric parameters (Å, º)

**Table:**

O1A—C11A	1.336 (2)	O6B—C14B	1.210 (3)
O1A—C7A	1.449 (2)	C7A—H7A	0.95 (2)
O3A—C24A	1.359 (3)	C6B—C5B	1.371 (3)
O3A—C18A	1.397 (3)	C6B—C1B	1.374 (3)
N1A—C11A	1.367 (3)	C21A—C20A	1.385 (4)
N1A—C14A	1.378 (3)	C21A—H21A	0.9300
N1A—C15A	1.471 (3)	C15A—H15A	0.9600
O6A—C14A	1.214 (3)	C15A—H15B	0.9600
O5A—C13A	1.218 (3)	C15A—H15C	0.9600
O1B—C11B	1.337 (3)	C15A—H15D	0.9600
O1B—C7B	1.455 (3)	C15A—H15E	0.9600
O2B—C24B	1.188 (3)	C15A—H15F	0.9600
O3B—C24B	1.354 (3)	C2B—C3B	1.359 (4)
O3B—C18B	1.396 (3)	C2B—C1B	1.374 (4)
O2A—C24A	1.186 (3)	C2B—H2B	0.9300
C11A—C10A	1.346 (3)	C16A—H16A	0.9600
N2A—C14A	1.376 (3)	C16A—H16B	0.9600
N2A—C13A	1.401 (3)	C16A—H16C	0.9600
N2A—C16A	1.469 (3)	C16A—H16D	0.9600
C9A—C10A	1.508 (3)	C16A—H16E	0.9600
C9A—C17A	1.515 (3)	C16A—H16F	0.9600
C9A—C8A	1.529 (3)	C1A—C2A	1.379 (4)
C9A—H9A	0.98 (2)	C1A—H1A	0.9300
C10A—C13A	1.434 (3)	C5B—C4B	1.382 (4)
C24A—C8A	1.506 (3)	C5B—H5B	0.9300
C17A—C18A	1.383 (3)	C20A—H20A	0.9300
C17A—C22A	1.386 (3)	C20B—C21B	1.374 (4)
O4A—C19A	1.357 (3)	C20B—C19B	1.374 (4)
O4A—C23A	1.426 (3)	C20B—H20B	0.9300
N1B—C11B	1.372 (3)	C16B—H16G	0.9600
N1B—C14B	1.383 (3)	C16B—H16H	0.9600
N1B—C15B	1.459 (4)	C16B—H16I	0.9600
O5B—C13B	1.219 (3)	C16B—H16J	0.9600
C6A—C1A	1.379 (3)	C16B—H16K	0.9600
C6A—C5A	1.380 (4)	C16B—H16L	0.9600
C6A—C7A	1.497 (3)	C22B—C21B	1.372 (4)
C11B—C10B	1.343 (3)	C22B—H22B	0.9300
N2B—C14B	1.368 (4)	C23A—H23A	0.9600
N2B—C13B	1.402 (3)	C23A—H23B	0.9600
N2B—C16B	1.463 (3)	C23A—H23C	0.9600
C8B—C24B	1.509 (3)	C4A—C3A	1.373 (5)
C8B—C9B	1.519 (3)	C4A—C5A	1.379 (4)
C8B—C7B	1.528 (3)	C4A—H4A	0.9300
C8B—H8B	0.99 (2)	C3A—C2A	1.359 (5)
C18A—C19A	1.391 (3)	C3A—H3A	0.9300
C10B—C13B	1.432 (3)	C2A—H2A	0.9300
C10B—C9B	1.505 (3)	C5A—H5A	0.9300
C18B—C17B	1.378 (3)	C1B—H1B	0.9300
C18B—C19B	1.392 (3)	C3B—C4B	1.371 (4)
O4B—C19B	1.359 (3)	C3B—H3B	0.9300
O4B—C23B	1.424 (3)	C23B—H23D	0.9600
C22A—C21A	1.374 (3)	C23B—H23E	0.9600
C22A—H22A	0.9300	C23B—H23F	0.9600
C8A—C7A	1.531 (3)	C15B—H15G	0.9600
C8A—H8A	0.97 (2)	C15B—H15H	0.9600
C9B—C17B	1.519 (3)	C15B—H15I	0.9600
C9B—H9B	0.99 (2)	C15B—H15J	0.9600
C7B—C6B	1.499 (3)	C15B—H15K	0.9600
C7B—H7B	1.06 (2)	C15B—H15L	0.9600
C19A—C20A	1.380 (4)	C21B—H21B	0.9300
C17B—C22B	1.375 (3)	C4B—H4B	0.9300
			
C11A—O1A—C7A	116.19 (16)	N1A—C15A—H15F	109.5
C24A—O3A—C18A	121.01 (17)	H15A—C15A—H15F	56.3
C11A—N1A—C14A	121.25 (18)	H15B—C15A—H15F	56.3
C11A—N1A—C15A	121.10 (18)	H15C—C15A—H15F	141.1
C14A—N1A—C15A	117.64 (18)	H15D—C15A—H15F	109.5
C11B—O1B—C7B	116.98 (17)	H15E—C15A—H15F	109.5
C24B—O3B—C18B	120.60 (17)	C3B—C2B—C1B	120.5 (3)
O1A—C11A—C10A	125.08 (19)	C3B—C2B—H2B	119.8
O1A—C11A—N1A	111.66 (17)	C1B—C2B—H2B	119.8
C10A—C11A—N1A	123.25 (19)	N2A—C16A—H16A	109.5
C14A—N2A—C13A	124.81 (18)	N2A—C16A—H16B	109.5
C14A—N2A—C16A	117.42 (19)	H16A—C16A—H16B	109.5
C13A—N2A—C16A	117.8 (2)	N2A—C16A—H16C	109.5
C10A—C9A—C17A	115.84 (19)	H16A—C16A—H16C	109.5
C10A—C9A—C8A	107.27 (16)	H16B—C16A—H16C	109.5
C17A—C9A—C8A	109.59 (17)	N2A—C16A—H16D	109.5
C10A—C9A—H9A	109.1 (12)	H16A—C16A—H16D	141.1
C17A—C9A—H9A	108.2 (12)	H16B—C16A—H16D	56.3
C8A—C9A—H9A	106.4 (13)	H16C—C16A—H16D	56.3
C11A—C10A—C13A	118.46 (19)	N2A—C16A—H16E	109.5
C11A—C10A—C9A	121.14 (19)	H16A—C16A—H16E	56.3
C13A—C10A—C9A	119.85 (19)	H16B—C16A—H16E	141.1
O2A—C24A—O3A	117.9 (2)	H16C—C16A—H16E	56.3
O2A—C24A—C8A	125.0 (2)	H16D—C16A—H16E	109.5
O3A—C24A—C8A	117.06 (19)	N2A—C16A—H16F	109.5
C18A—C17A—C22A	117.7 (2)	H16A—C16A—H16F	56.3
C18A—C17A—C9A	118.41 (19)	H16B—C16A—H16F	56.3
C22A—C17A—C9A	123.88 (19)	H16C—C16A—H16F	141.1
C19A—O4A—C23A	117.9 (2)	H16D—C16A—H16F	109.5
C11B—N1B—C14B	121.2 (2)	H16E—C16A—H16F	109.5
C11B—N1B—C15B	121.0 (2)	C6A—C1A—C2A	120.1 (3)
C14B—N1B—C15B	117.8 (2)	C6A—C1A—H1A	120.0
O6A—C14A—N2A	122.7 (2)	C2A—C1A—H1A	120.0
O6A—C14A—N1A	121.3 (2)	C6B—C5B—C4B	120.4 (3)
N2A—C14A—N1A	116.01 (19)	C6B—C5B—H5B	119.8
C1A—C6A—C5A	119.2 (2)	C4B—C5B—H5B	119.8
C1A—C6A—C7A	119.3 (2)	C19A—C20A—C21A	119.7 (2)
C5A—C6A—C7A	121.5 (2)	C19A—C20A—H20A	120.1
O1B—C11B—C10B	125.2 (2)	C21A—C20A—H20A	120.1
O1B—C11B—N1B	112.0 (2)	C21B—C20B—C19B	119.8 (2)
C10B—C11B—N1B	122.8 (2)	C21B—C20B—H20B	120.1
C14B—N2B—C13B	125.3 (2)	C19B—C20B—H20B	120.1
C14B—N2B—C16B	116.9 (2)	O4B—C19B—C20B	125.5 (2)
C13B—N2B—C16B	117.9 (2)	O4B—C19B—C18B	116.3 (2)
C24B—C8B—C9B	112.13 (18)	C20B—C19B—C18B	118.2 (2)
C24B—C8B—C7B	111.53 (19)	N2B—C16B—H16G	109.5
C9B—C8B—C7B	108.64 (19)	N2B—C16B—H16H	109.5
C24B—C8B—H8B	105.6 (14)	H16G—C16B—H16H	109.5
C9B—C8B—H8B	112.2 (14)	N2B—C16B—H16I	109.5
C7B—C8B—H8B	106.6 (14)	H16G—C16B—H16I	109.5
C17A—C18A—C19A	122.6 (2)	H16H—C16B—H16I	109.5
C17A—C18A—O3A	122.64 (19)	N2B—C16B—H16J	109.5
C19A—C18A—O3A	114.73 (19)	H16G—C16B—H16J	141.1
C11B—C10B—C13B	119.1 (2)	H16H—C16B—H16J	56.3
C11B—C10B—C9B	120.6 (2)	H16I—C16B—H16J	56.3
C13B—C10B—C9B	120.0 (2)	N2B—C16B—H16K	109.5
C17B—C18B—C19B	122.3 (2)	H16G—C16B—H16K	56.3
C17B—C18B—O3B	122.6 (2)	H16H—C16B—H16K	141.1
C19B—C18B—O3B	115.0 (2)	H16I—C16B—H16K	56.3
C19B—O4B—C23B	117.3 (2)	H16J—C16B—H16K	109.5
C21A—C22A—C17A	120.5 (2)	N2B—C16B—H16L	109.5
C21A—C22A—H22A	119.7	H16G—C16B—H16L	56.3
C17A—C22A—H22A	119.7	H16H—C16B—H16L	56.3
O5B—C13B—N2B	119.9 (2)	H16I—C16B—H16L	141.1
O5B—C13B—C10B	124.5 (2)	H16J—C16B—H16L	109.5
N2B—C13B—C10B	115.6 (2)	H16K—C16B—H16L	109.5
C24A—C8A—C9A	112.47 (18)	C21B—C22B—C17B	120.2 (3)
C24A—C8A—C7A	111.13 (18)	C21B—C22B—H22B	119.9
C9A—C8A—C7A	108.06 (18)	C17B—C22B—H22B	119.9
C24A—C8A—H8A	107.2 (13)	O4A—C23A—H23A	109.5
C9A—C8A—H8A	110.6 (13)	O4A—C23A—H23B	109.5
C7A—C8A—H8A	107.3 (13)	H23A—C23A—H23B	109.5
O2B—C24B—O3B	117.7 (2)	O4A—C23A—H23C	109.5
O2B—C24B—C8B	124.5 (2)	H23A—C23A—H23C	109.5
O3B—C24B—C8B	117.79 (19)	H23B—C23A—H23C	109.5
C10B—C9B—C8B	108.28 (18)	C3A—C4A—C5A	120.4 (3)
C10B—C9B—C17B	115.05 (19)	C3A—C4A—H4A	119.8
C8B—C9B—C17B	109.41 (18)	C5A—C4A—H4A	119.8
C10B—C9B—H9B	108.0 (13)	C2A—C3A—C4A	119.7 (3)
C8B—C9B—H9B	106.7 (13)	C2A—C3A—H3A	120.2
C17B—C9B—H9B	109.1 (13)	C4A—C3A—H3A	120.2
O1B—C7B—C6B	106.77 (18)	C3A—C2A—C1A	120.7 (3)
O1B—C7B—C8B	108.42 (18)	C3A—C2A—H2A	119.7
C6B—C7B—C8B	114.8 (2)	C1A—C2A—H2A	119.7
O1B—C7B—H7B	109.9 (13)	C6A—C5A—C4A	120.0 (3)
C6B—C7B—H7B	109.9 (13)	C6A—C5A—H5A	120.0
C8B—C7B—H7B	107.0 (13)	C4A—C5A—H5A	120.0
O4A—C19A—C20A	125.6 (2)	C2B—C1B—C6B	120.8 (3)
O4A—C19A—C18A	116.1 (2)	C2B—C1B—H1B	119.6
C20A—C19A—C18A	118.4 (2)	C6B—C1B—H1B	119.6
C22B—C17B—C18B	118.1 (2)	C2B—C3B—C4B	119.4 (3)
C22B—C17B—C9B	124.3 (2)	C2B—C3B—H3B	120.3
C18B—C17B—C9B	117.61 (19)	C4B—C3B—H3B	120.3
O5A—C13A—N2A	119.7 (2)	O4B—C23B—H23D	109.5
O5A—C13A—C10A	124.3 (2)	O4B—C23B—H23E	109.5
N2A—C13A—C10A	116.00 (19)	H23D—C23B—H23E	109.5
O1A—C7A—C6A	106.58 (17)	O4B—C23B—H23F	109.5
O1A—C7A—C8A	107.71 (17)	H23D—C23B—H23F	109.5
C6A—C7A—C8A	116.74 (19)	H23E—C23B—H23F	109.5
O1A—C7A—H7A	107.5 (14)	N1B—C15B—H15G	109.5
C6A—C7A—H7A	108.4 (13)	N1B—C15B—H15H	109.5
C8A—C7A—H7A	109.5 (14)	H15G—C15B—H15H	109.5
O6B—C14B—N2B	122.6 (3)	N1B—C15B—H15I	109.5
O6B—C14B—N1B	121.3 (3)	H15G—C15B—H15I	109.5
N2B—C14B—N1B	116.0 (2)	H15H—C15B—H15I	109.5
C5B—C6B—C1B	118.6 (2)	N1B—C15B—H15J	109.5
C5B—C6B—C7B	122.5 (2)	H15G—C15B—H15J	141.1
C1B—C6B—C7B	118.8 (2)	H15H—C15B—H15J	56.3
C22A—C21A—C20A	121.0 (2)	H15I—C15B—H15J	56.3
C22A—C21A—H21A	119.5	N1B—C15B—H15K	109.5
C20A—C21A—H21A	119.5	H15G—C15B—H15K	56.3
N1A—C15A—H15A	109.5	H15H—C15B—H15K	141.1
N1A—C15A—H15B	109.5	H15I—C15B—H15K	56.3
H15A—C15A—H15B	109.5	H15J—C15B—H15K	109.5
N1A—C15A—H15C	109.5	N1B—C15B—H15L	109.5
H15A—C15A—H15C	109.5	H15G—C15B—H15L	56.3
H15B—C15A—H15C	109.5	H15H—C15B—H15L	56.3
N1A—C15A—H15D	109.5	H15I—C15B—H15L	141.1
H15A—C15A—H15D	141.1	H15J—C15B—H15L	109.5
H15B—C15A—H15D	56.3	H15K—C15B—H15L	109.5
H15C—C15A—H15D	56.3	C22B—C21B—C20B	121.3 (3)
N1A—C15A—H15E	109.5	C22B—C21B—H21B	119.3
H15A—C15A—H15E	56.3	C20B—C21B—H21B	119.3
H15B—C15A—H15E	141.1	C3B—C4B—C5B	120.2 (3)
H15C—C15A—H15E	56.3	C3B—C4B—H4B	119.9
H15D—C15A—H15E	109.5	C5B—C4B—H4B	119.9

### Hydrogen-bond geometry (Å, º)

**Table:**

*D*—H···*A*	*D*—H	H···*A*	*D*···*A*	*D*—H···*A*
C21*A*—H21*A*···O5*B*^i^	0.93	2.55	3.435 (4)	159
C20*B*—H20*B*···O5*A*^ii^	0.93	2.34	3.175 (3)	149
C23*B*—H23*D*···O6*B*^iii^	0.96	2.46	3.249 (4)	139

Symmetry codes: (i) −*x*+1, −*y*+2, −*z*+1; (ii) −*x*+2, −*y*+2, −*z*+1; (iii) *x*, *y*−1, *z*.
